# Spatial distribution of isobaric androgens in target tissues using chemical derivatization and MALDI-2 on a trapped ion mobility quadrupole time-of-flight instrument†

**DOI:** 10.1039/d1ra06086d

**Published:** 2021-10-18

**Authors:** C. L. Logan Mackay, Jens Soltwisch, Bram Heijs, Karl W. Smith, Faye L. Cruickshank, Annika Nyhuis, Klaus Dreisewerd, Diego Cobice

**Affiliations:** SIRCAMS, EastChem School of Chemistry, University of Edinburgh Scotland UK; Institute of Hygiene, University of Münster Münster Germany; Mass Spectrometry Imaging Group, Leids Universitair Medisch Centrum Leiden The Netherlands; Center for Proteomics & Metabolomics, Leiden University Medical Centre Leiden The Netherlands; Mass Spectrometry Centre, Biomedical Science Research Institute (BMSRI), Ulster University Coleraine Northern Ireland UK d.cobice@ulster.ac.uk +44 (0)2892 604456; Bruker Daltonics GmbH & Co. KG Bremen Germany

## Abstract

Prostate cancer is initially treated *via* androgen deprivation therapy (ADT), a highly successful treatment in the initial pursuit of tumour regression, but commonly restricted by the eventual emergence of a more lethal ‘castrate resistant’ (CRPC) form of the disease. Intracrine pathways that utilize dehydroepiandrosterone (DHEA) or other circulatory precursor steroids are thought to generate relevant levels of growth-stimulating androgens such as testosterone (T) and dihydrotestosterone (DHT). Decoding this tissue-specific metabolic pathway is key for the development of novel therapeutic treatments. Mass spectrometry imaging (MSI) is an analytical technique that allows the visualization of the distribution of numerous classes of biomolecules within tissue sections. The analysis of androgens by liquid chromatography mass spectrometry (LC/MS)-based methods however presents a challenge due to their generally poor ionization efficiency and low physiological endogenous levels. In MSI, on-tissue chemical derivatization (OTCD) has enabled the limits of steroids to be imaged within tissues to be pushed by overcoming poor ionization performance. However, isobaric interference of key androgen derivatives such as T and DHEA can severely hamper studying the intracrinology in several diseases. Here, we have evaluated the use of laser induced post-ionization (MALDI-2) combined with trapped ion mobility separation (TIMS) and orthogonal time-of-flight (QTOF) MS for the visualization of isobaric derivatized androgens in murine tumour xenograft at about 50 μm spatial resolution. With this combination, isobaric T and DHEA were separated in tissue sections and the signals of derivatized steroids enhanced by about 20 times. The combination of TIMS and MALDI-2 thus shows unique potential to study tissue intracrinology within target tissues. This could offer the opportunity for many novel insights into tissue-specific androgen biology.

## Abstract 2

We increase ionization efficiency and isobaric separation of derivatized androgens using MALDI-2-TIMS. First time spatial distribution of isobaric androgens is achieved in biological tissues by on-tissue chemical derivatization and MALDI-2-TIMS-MSI.

## Graphical abstract

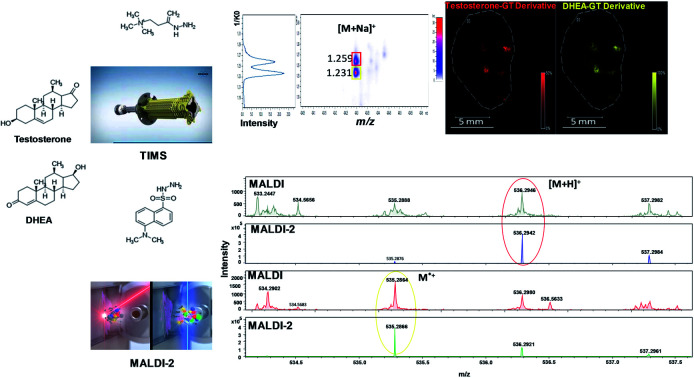

## Introduction

**Fig. 1:**
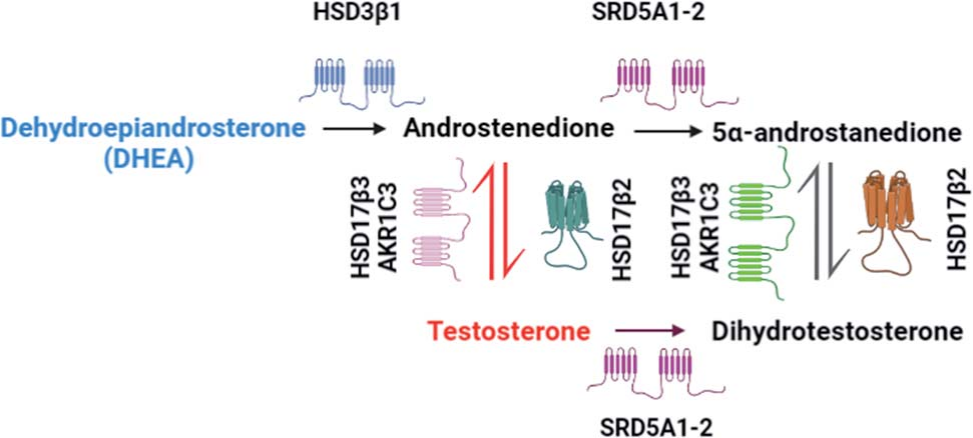
Main castrate resistant prostate cancer (CRPC) androgen pathway. Adapted from Chang *et al.*^1^

Advanced localized prostate cancer (PCa) is commonly treated by androgen deprivation therapy (ADT).^1^ The primary goal of this treatment is to decrease the levels of circulatory androgens, such as testosterone (T) and to block tumour cell growth. Castrate resistant prostate cancer (CRPC) is a more lethal and difficult form of PCa to treat and it is currently under debate on how this alternative form has originated.^1–3^ Steroid intracrinology was first acknowledged by Labrie *et al.*,^4^ in which castrated rats obtained the ability to maintain levels of T and dihydrotestosterone (DHT). These potent androgen receptor activating ligands, by conversion mechanisms contained within the prostatic tumour tissue, are synthesized using the adrenal precursor dehydroepiandrosterone (DHEA).^5^ The retention of androgen signalling has been suggested upon this mechanism of intracrine conversion, with numerous studies showing the presence of a different androgen metabolism pathway as shown in Fig. 1.^1,5^ Therefore, unravelling this tissue-specific metabolic pathway is key for the development of novel therapeutic treatments.

Mass spectrometry imaging (MSI) is a versatile and multiplex analytical method, allowing the production of a 2D ion image of multiple metabolites in biological tissues.^6^ Androgens have been demonstrated to show poor ionization efficiency in liquid chromatography/mass spectrometry (LC/MS) analysis due to the lack of both hydrogen donor or acceptor moieties. Moreover, their physiological endogenous levels are generally low.^7^ Chemical derivatization (CD) approaches have been extensively used in LC/MS-based platforms in order to enhance ion production yields. In combination with matrix-assisted laser desorption ionization (MALDI) MSI, on-tissue chemical derivatization (OTCD) has been able to push the limits of steroid imaging within tissues by increasing signal intensity and overcoming poor ionization performance.^8,9^ Previous studies have demonstrated the use of hydrazine-based derivatization reagents in androgen MSI for murine tissue sections.^10,11^ However, isobaric interference of key derivatized androgens such as T and DHEA, along with ion suppression effects, remain an issue as both androgen derivatives share the same monoisotopic mass and the same fragmentation patterns upon several dissociation mechanisms.^10^

To overcome these issues, we here assessed, for the first time, the use of laser-induced post-ionization (MALDI-2), combined with trapped ion mobility separation (TIMS) (MALDI-2-TIMS) in the identification and distribution analysis of isobaric derivatized androgen in a murine tumour xenograft model. MALDI-2 is a recently introduced method for enhanced MSI of numerous classes of biomolecules including sterols,^12^ in tissue sections at high lateral resolution.^13^

In this proof of concept study, two derivatization reagents, namely Girard-T (GT) and Dansyl Hydrazine (DS) were evaluated using a timsTOF fleX MALDI-2 platform from Bruker. The laser-induced post-ionization process significantly improved sensitivity of the derivatized androgens and TIMS provided fast orthogonal separation that efficiently unravels isobaric androgen spectra. The result is not only the ability to extract mass information from single isobars, but also to differ isobars based on their different collisional cross sections (CCS) and hence ion mobility drift times. In this way, the spatial distribution of both DHEA and T in a murine xenograft model was achieved.

## Results and discussion

### Off-tissue derivatization screening and TIMS separation

This novel application of OTCD coupled with MALDI-2 and TIMS enhanced on-tissue detection by laser induced post-ionization on readily charged androgen derivatives and allowed separation of endogenous isobaric androgen derivatives, which yielded intense signal and distinct gas-phase conformations upon TIMS analysis.

In this study, two hydrazine-based derivatization reagents were evaluated using previously described optimized reaction conditions^8–10^ to investigate the MALDI-2 signal enhancement and TIMS mobility separation performance (Fig. S1†). These reagents (Girard-T (GT) and Dansyl Hydrazine (DS)) (Fig. S1†) were screened off and on-tissue, and instrument parameters were optimized.

As shown in Fig. S2a,† testosterone GT-derivative (GT-T) was detected at *m*/*z* 402.3138 as a molecular ion (M^+^) with a mass accuracy (deviation from the calculated mass) of 5.7 ppm and a signal intensity of 5 × 10^6^. Its sodium adduct was also observed at *m*/*z* 424.2947 [M + Na]^+^ with a mass accuracy of 2.8 ppm and a signal intensity of 5.5 × 10^5^. Fig. S2b† displays dehydroepiandrosterone GT-DHEA detected at *m*/*z* 402.3143 (M^+^) with a mass accuracy of 6.9 ppm and a signal intensity of 6 × 10^6^. Its sodium adduct was also detected at *m*/*z* 424.2946 [M + Na]^+^ with a mass accuracy of 2.6 ppm and a signal intensity of 1 × 10^6^.

That both androgen derivatives displayed a sodium adduct is an interesting finding since these charged derivatives possess a quaternary amine moiety and sodium adduct formation therefore was not expected. Also, the detection of these ion species was not reported previously in the MALDI analysis of hydrazone derivatives. The adduct formation mechanism may be explained by the thermal proton transfer model,^14^ in which the absorption of laser light by matrix molecules results in an increased temperature, upon which the solid matrix melts and behaves in a similar manner to a polar solvent, before the occurrence of desorption. Adducts may then be generated by thermally induced reactions in a polar solvent-like matrix by proton transfer from the quaternary amine.

On the other hand, as is shown in Fig. S2c,† testosterone DS derivative (DS-T) displays a protonated mass at *m*/*z* 536.2942 [M + H]^+^ with a mass accuracy of 0.2 ppm and a signal intensity of 4 × 10^5^. Sodiated adduct was also found at *m*/*z* 558.2757 [M + Na]^+^ with a mass accuracy of −0.7 ppm and a signal intensity of 2 × 10^5^. The DHEA-DS derivative (DS-DHEA) was also detected (Fig. S2d†) as a radical cation at *m*/*z* 535.2868 (M^+^˙) with a mass accuracy of 0.9 ppm at 3.5 × 10^5^ signal intensity and as a sodium adduct at *m*/*z* 558.2750 [M + Na]^+^ with a mass accuracy of −2.0 ppm and a signal intensity of 1.5 × 10^5^.

It is important to note that even though both androgen derivatives can be resolved by mass, as DS-T is detected as protonated mass and DS-DHEA as radical cation, DS-T derivative may suffer from isobaric interference from the ^13^C isotope peak of DS-DHEA unless using ultra-high mass resolution analysers such as FT-ICR. The different behaviour of DS-derivatives regarding pseudomolecular ion formation may be attributable to their molecular structure. The protonation site of DS-T derivative could be either at the nitrogen of tertiary amine or hydrazine groups. In both scenarios the charge can be stabilized by the π–π system which, in case of testosterone, is extended to the steroid A ring due to its α,β-unsaturated carbonyl system at C4. Since DHEA-DS lacks this conjugated system, protonation is less energetically favoured, thus a radical cation may be formed by losing one electron from either the hydrazine or tertiary amine nitrogen by direct photo/thermal mechanism during the ionization process.

**Fig. 2:**
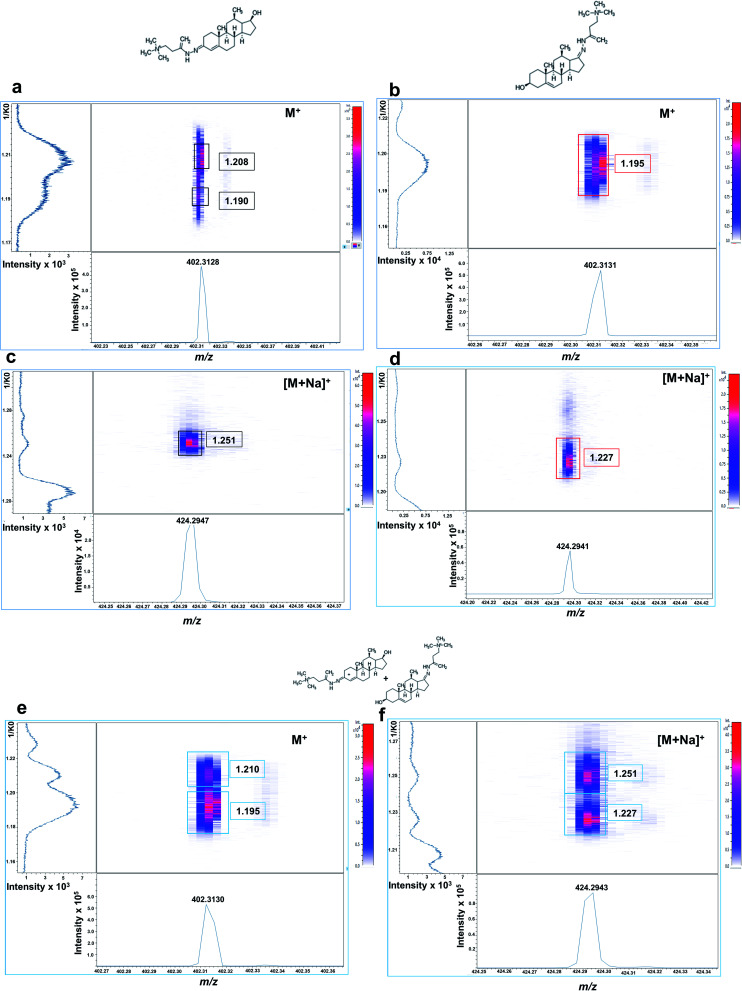
Off-tissue mobility assessment of Girard-T-derivatized androgens by MALDI-2-TIMS-tof mass spectrometry. Mobilograms of derivatized (a) testosterone (M^+^ signal at *m*/*z* 402.3138) showing two ion mobility signals with central 1/*K*_0_ values at 1.190 and 1.208, respectively. (b) DHEA M^+^ signal at *m*/*z* 402.3143 showing a single ion mobility signal at 1.195 1/*K*_0_. (c) Testosterone ([M + Na]^+^ at *m*/*z* 424.2947) showing a single ion mobility signal at 1.251 1/*K*_0_. (d) DHEA ([M + Na]^+^ at *m*/*z* 424.2946) showing a single ion mobility signal at 1.227 1/*K*_0_. (e) Equimolar solution of isobaric T and DHEA (100 ng mL^−1^, each) showing two ion mobility signals at 1.210 1/*K*_0_ (T) and 1.195 1/*K*_0_ (DHEA) for the at M^+^ ion. (f) Data from the same equimolar solution for the [M + Na]^+^ species of T and DHEA showing two ion mobility signals at 1.251 1/*K*_0_ (T) and 1.227 1/*K*_0_ (DHEA). Signal intensity is depicted by colour on the scales shown.

Isobaric GT-androgen derivatives were then subjected to TIMS separation based on their CCS. As shown in Fig. 2a, GT-T derivative exhibited two mobility signals at 1/*K*_0_ values of 1.208 and 1.190, presumably corresponding to its *cis*–*trans* isomeric structures across the enamine moiety double bond (C=N) on the A ring at C3. In contrast, the DHEA-GT derivative displayed only one mobility signal at 1.195 1/*K*_0_ (Fig. 2b) as the two isomeric structures could not be resolved, probably due to possible steric hindrance effects from the methyl group near the enamine moiety on the D ring at C17. Their sodiated adducts were also assessed and, interestingly displayed high, intensely distinct mobility signals at 1.251 1/*K*_0_ for GT-T (Fig. 2c) and 1.227 1/*K*_0_ for GT-DHEA (Fig. 2d).

If analysed from an equimolar mixture, the two isobaric androgen derivatives were resolved at M^+^ at 1.210 1/*K*_0_ for GT-T and 1.195 1/*K*_0_ for GT-DHEA (Fig. 2e) and at [M + Na]^+^ at 1.251 for GT-T and 1.227 for GT-DHEA with better resolution observed for the sodium adducts (Fig. 2f). This data suggested that there is a potential overlap of one GT-T isomer at 1.190 1/*K*_0_ with the GT-DHEA at 1.195 1/*K*_0_ at M^+^.

**Fig. 3:**
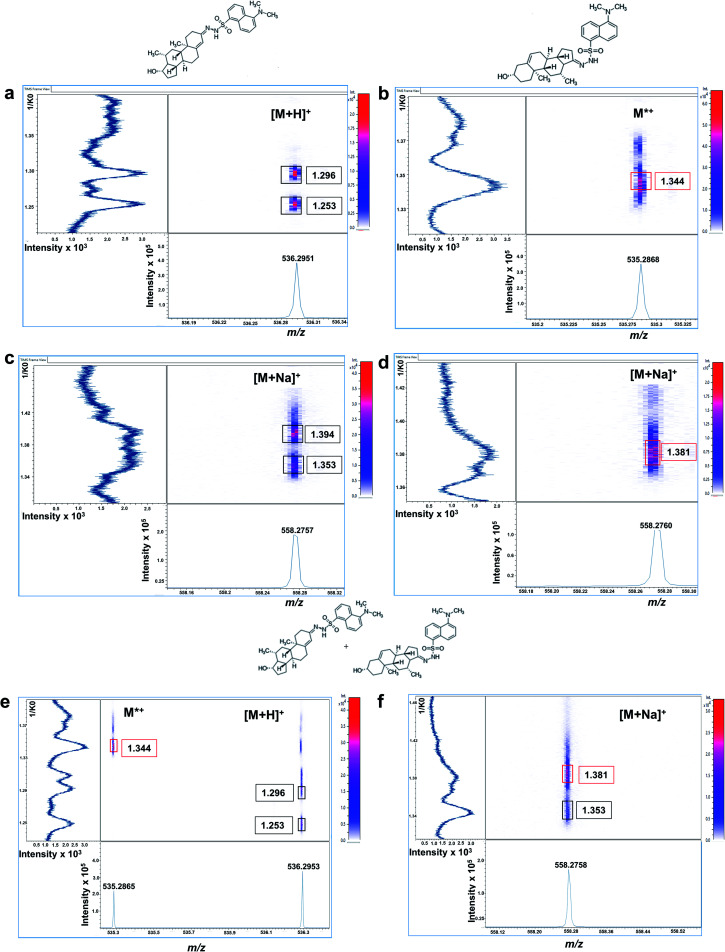
Off-tissue mobility assessment of dansyl hydrazine-derivatized androgens by MALDI-TIMS-tof mass spectrometry. Mobilograms of derivatized (a) testosterone [M + H]^+^ signal at *m*/*z* 536.2942 showing two ion mobility signals with central 1/*K*_0_ values at 1.296 and 1.253, respectively. (b) DHEA M^+^* signal at *m*/*z* 535.2868 showing a single ion mobility signal at 1.344 1/*K*_0_. (c) Testosterone ([M + Na]^+^ at *m*/*z* 558.2750) showing two ion mobility signals at 1.394 and 1.353 1/*K*_0_. (d) DHEA ([M + Na]^+^ at *m*/*z* 558.2750) showing a single ion mobility signal at 1.381 1/*K*_0_. (e) Equimolar solution of isobaric T and DHEA (100 ng mL^−1^, each) [M + H]^+^ at *m*/*z* 536.2940 (T) showing a single ion mobility signal at 1.344 1/*K*_0_ (T) and M^+^* (DHEA) at *m*/*z* 535.2865 showing two ion mobility signals with central 1/*K*_0_ values at 1.296 and 1.253 (DHEA), respectively. (f) Data from the same equimolar solution for the [M + Na]^+^ species of T and DHEA showing two ion mobility signals at 1.353 1/*K*_0_ (T) and 1.381 1/*K*_0_ (DHEA). Signal intensity is depicted by colour on the scales shown.

Mobilograms for dansyl derivatives are shown in Fig. 3. Protonated DS-T derivative exhibited two mobility signals at 1.296 and 1.253 1/*K*_0_ corresponding to its corresponding *cis*–*trans* isomeric structures, as previously observed for GT-derivatives (Fig. 3a). The radical DS-DHEA derivative only shows one intense mobility signal at 1.344 1/*K*_0_ (Fig. 3b). Regarding their sodium adducts, DS-T displayed two mobility signals at 1.394 and 1.353 1/*K*_0_ with only one signal observed for DS-DHEA at 1.381 1/*K*_0_, as shown in Fig. 3c and d.

From an equimolar mixture the two androgens derivatives were successfully resolved at [M + H]^+^ at 1.296/1.253 1/*K*_0_ for DS-T and at M^+^˙1.344 1/*K*_0_ for DS-DHEA (Fig. 3e). They were also resolved at [M + Na]^+^ mobility signals detected at 1.353 for GT-T and 1.381 1/*K*_0_ for GT-DHEA (Fig. 3f), with a better mobility resolution observed for the protonated/radical ions.

### On-tissue derivatisation screening and MALDI-2-TIMS-tof MSI assessment

Signal enhancement upon laser-induced post-ionization was evaluated on tumour xenograft tissue, in which the levels of endogenous tumour T and DHEA are high (around 20 ng g^−1^) as determined by LC/MS. Tissue sections were derivatized using both GT and DS reagents as described in the methods section.

**Fig. 4:**
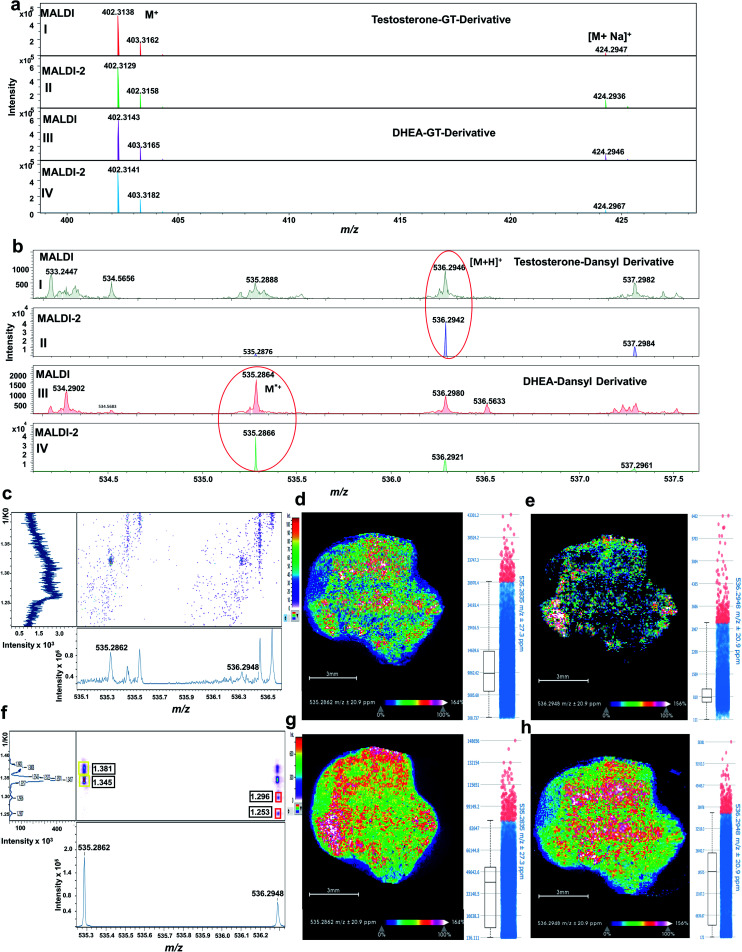
OTCD-MALDI-2-TIMS-tof MS imaging analysis of endogenous androgens as hydrazone derivatives in LNCaP tumour control tissue from a xenograft mouse model. (a) MALDI-2-TIMS-tof MS analysis (accumulation of 20 random walk scans across the tissue) of GT-derivatives of (I) testosterone with MALDI-2 disabled, (IV) testosterone with MALDI-2 enabled, (III) DHEA with MALDI-2 disabled, and (IV) DHEA MALDI-2 enabled. For these derivatives, signal intensity was not enhanced upon activation of laser-induced post-ionization. (b) MALDI-2-tof MSI analysis of DS-derivatives. Signal intensity was considerably enhanced upon use of laser post-ionization: (I) testosterone measured with MALDI-2 disabled, (II) testosterone with MALDI-2 enabled. (III) DHEA with MALDI-2 disabled, and (IV) DHEA with MALDI-2 enabled. Mobilograms of DS-T and DS-DHEA derivatives: (c) without MALDI-2, (f) with MALDI-2 enabled. MSI image (whole tissue) reconstruction along with box-plot signal intensity for the DS-DHEA derivative (M^+^˙) at *m*/*z* 535.2862 (sum of *cis*–*trans* isomers at 1.381 and 1.345 1/*K*_0_): (d) without MALDI-2 enabled, (g) with MALDI-2 enabled. DS-T [M + H]^+^ at *m*/*z* 536.2948 (sum of *cis*–*trans* isomers at 1.296 and 1.253 1/*K*_0_): (e) without MALDI-2, (h) with MALDI-2 enabled. Signal intensity is depicted by colour on the scales shown. Scale bar, 3 mm.

As clearly shown in Fig. 4a, GT-derivatives did not exhibit any signal enhancement upon MALDI-2. Since GT derivatives are already charged species, they apparently do not benefit from the MALDI-2 process. Similar observations have been made previously for example for quaternary phosphatidylcholines.^15,16^ On the contrary, signal intensity of DS-derivatives (accumulation of 20 random walk scans across the tissue) was enhanced upon MALDI-2 by about 20 times for DS-DHEA and 40 times for DS-T derivatives (Fig. 4b).

Since DS-derivatives are not charged species, they are more susceptible to ion suppression effects than GT-charged derivatives. In this case, MALDI-2 has substantially improved on-tissue signal intensity of DS-androgen derivatives.

The distributions of the endogenous isobaric androgens in the tissue was also assessed with and without MALDI-2. Signal of endogenous DS androgen derivatives without laser-induced post-ionization was considerable reduced as signal intensity for both DS derivatives were suppressed; for the DS-T derivative, the signal was almost undetectable as displayed in Fig. 4c. As a result, the MSI image of DS-DHEA at *m*/*z* 535.2862 (sum of *cis*–*trans* isomers at 1.381 and 1.345 1/*K*_0_) and DS-T at *m*/*z* 536.2948 (sum of *cis*–*trans* isomers at 1.296 and 1.253 1/*K*_0_) displayed low signal intensity, as shown in Fig. 4d and e, respectively. Upon enabling MALDI-2, tissue signal on the selected region of interest (ROI) was greatly improved, as shown in Fig. 4f. Consequently, MSI images signal intensities of DS-DHEA at *m*/*z* 535.2862 and DS-T at *m*/*z* 536.2948 were enhanced by approximately 5 times for DS-DHEA and by about 20 times for DS-T, as is shown in Fig. 4g and h respectively.

### Distribution of endogenous T and DHEA in LNCaP mouse xenograft tissues by OTCD-MALDI-2-TIMS-tof

For the first time, isobaric T and DHEA were successfully detected and resolved, and their spatial distribution was assessed in murine xenograft tissues using the OTCD-MALDI-2-TIMS-tof MSI platform. Understanding the distribution of these androgens in the prostate tumour is strategic at aiming to establish how potential effects of a tumour environment may have on the intracrine pathway of steroidogenesis. It is also key to design therapeutic treatment that may cause desirable effects to reduce the formation of cancer cells.

For molecular mapping of these androgens, GT was selected as derivatization reagent, because this achieved the best signal intensity and well-resolved mobilograms using sodium adducts for both T and DHEA derivatives.

**Fig. 5:**
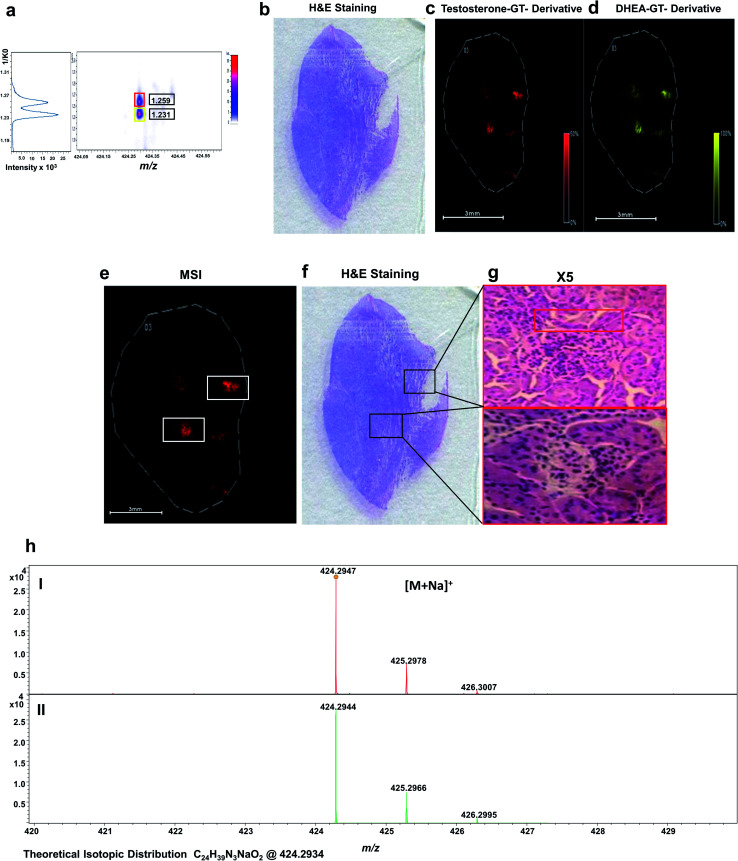
OTCD-MALDI-TIMS-tof MS imaging of androgens as Girard-T hydrazone derivatives of LNCaP tumour control tissue from a xenograft mouse. Molecular distribution of endogenous isobaric androgens detected as GT-derivatives in an LNCaP tumour control tissue from a xenograft mouse section by OTCD-MALDI-TIMS-tof-MSI platform. (a) Representative single-pixel mobilograms of isobaric androgens: GT-derivatives ([M + Na]^+^ at *m*/*z* 424.2940) detected at 1.259 1/*K*_0_ for testosterone and at 1.231 1/*K*_0_ for DHEA. (b) Representative H&E-stained adjacent tissue section. Molecular distribution map of (c) GT-T derivative ([M + Na]^+^, *m*/*z* 424.2940 ± 0.005, 1.251 1/*K*_0_) (d) GT-DHEA derivative ([M + Na]^+^, *m*/*z* 424.2940 ± 0.005, 1.231 1/*K*_0_). (e) Molecular distribution map of GT-testosterone derivative ([M + Na]^+^ at *m*/*z* 424.2940, 1.259 1/*K*_0_) with high ion abundance areas highlighted. (f) Representative H&E-stained tissue with high ion abundance areas highlighted. (g) Magnified image obtained with ×5 zoom of areas indicated; (h) representative single pixel isobaric sodium adduct mass spectrum of (I) GT-T derivative, (II) GT-DHEA derivative. Signal intensity is depicted by colour on the scales shown. Scale bar, 3 mm.

As shown in Fig. 5, endogenous T and DHEA were detected as GT derivatives at *m*/*z* 424.2947, corresponding to the [M + Na]^+^ species with a mass accuracy of 2.3 ppm (Fig. 5i). T and DHEA were successful resolved by their distinct CCS of their sodium adducts at 1.259 1/*K*_0_ and 1.231 1/*K*_0_, respectively, as shown in Fig. 5a. Spatial distributions shown discrete localization within the tumour tissue with perfect co-localization were observed for both steroids (Fig. 5c and d).

Histological staining with haematoxylin and eosin (H&E) (Fig. 5b) was used to assess if any distinct morphological regions or differential cell types could be observed in the tissue that may be related to the intracrine conversion of androgens. The distributions of T and DHEA showed potential areas of differentiation, as indicated in Fig. 5e and f. A magnification is indicated up to 5× on the tissue regions of interest indicated in Fig. 5g.

Upon magnification, blood cells were distinguished from the mesenchymal stroma cells present, displaying nuclei stained as the darker coloured purple spots at 5×. Basal cells of the stroma are also observed as the uniform cells bordering the darker tissue staining of the blood vessels (highlighted in red). The blood vessels were less uniform and looked disordered within the tissue stains. No further differentiation of cell types was observed at these potential sites, and no distinct cell types were observed when correlating them with derivatized androgen signal detected. To confirm the identification of both active androgens in the tumour tissue, LC/MS was performed on tissue homogenate, giving a concentration of 31 pg g^−1^ for T and 52 pg g^−1^ for DHEA (Fig. S5†).

## Conclusions

OTCD-MALDI-2-TIMS-tof MSI is a powerful new tool to study the spatial distribution of derivatized isobaric androgens within tissues. Here, we have demonstrated the ability of the method for visualization of testosterone and DHEA, two biological exceedingly relevant androgens. We have also demonstrated the utility of the method for detecting endogenous concentrations within murine xenograft tissue, a key tissue model for prostate cancer intracrinology at 50 μm spatial resolution. Our proof of principle study strongly benefitted from the use of MALDI-2, as this technology not only enables the detection of native steroids, but advantageously also enhances the analytical sensitivity of more readily ionizable androgens derivatives by about five times and by reducing ion suppression effects. The additional TIMS unit, available with the employed mass spectrometer, provided fast orthogonal separation of the ions according to their individual CCS values. This feature efficiently unravelled these isobaric spectra. The combination of TIMS and MALDI-2 has shown to be a uniquely powerful platform to study tissue intracrinology within target tissues.

This offers the opportunity for many novel insights into tissue-specific androgens biology. Importantly, the use of this platform facilitates the analysis of previously inaccessible biologically relevant isobaric steroids through adaptation of existing chemical derivatisation methods. This platform may be applicable to other carbonyl containing molecules such as aldehydes, enolates and also amides/esters, in which case, will be feasible prior carbonyl activation. This technique may also be extended to study other poor ionisable carbonyl-containing molecules in biological relevant tissues such as kidney, brain and adrenal gland.

## Experimental

### Chemicals

Testosterone and dehydroepiandrosterone (DHEA) were purchased separately as 1 mg mL^−1^ solutions in methanol from Steraloids Inc. (Newport, RI); d_3_-testosterone (ISTD for LC/MS) was purchased form Sigma Aldrich (Dorset, UK). Girard-T (GT) reagent, Dansyl Hydrazine (DS), α-cyano-4-hydroxycinnamic acid (CHCA), acetonitrile, methanol, acetone, ethanol, trifluoroacetic acid (TFA) and HPLC grade water were all obtained from Sigma Aldrich (Dorset, UK).

### Cell line and culture conditions

LNCaP cell line was obtained from the European Collection of Authenticated Cell Cultures (LNCaP clone FGC (ECACC 89110211), Salisbury, UK) and maintained in RPMI1640 media with 10% foetal bovine serum (FBS), 1% penicillin/streptomycin and 1% l-glutamine (Fisher Scientific, Loughborough, UK). Cells were kept at 37 °C in a 5% CO_2_ environment.

### *In vivo* LNCaP mouse xenograft model

Experiments were conducted in accordance with the Animal (Scientific Procedures) Act 1986. All animal procedures were performed in accordance with the Guidelines for Care and Use of Laboratory Animals of Ulster University and approved by the Animal Ethics Committee of Ulster University. Male nude CD-1 mice (aged 6–8 weeks, weighing approximately 27–34 g) were housed at room temperature of 20–24 °C, with a 12 h light/dark cycle. Animals were housed in individually ventilated cages with up to 5 mice per cage, with standard certified diet food and water ad libitum. LNCaP human prostate cancer cells (1 × 10^7^ suspended in Matrigel) were implanted subcutaneously onto the flank of the mice using a 25-gauge needle. When the tumour volume reached approximately 100–150 mm^3^, mice were subjected to treatment. Mice were dosed with DHEA (dissolved in vehicle solution) at 0.5 μg kg^−1^ and administered by intraperitoneal route (IP). All surgical procedures were performed under aseptic conditions, and the body temperature of the animals was kept constant using heated pads. Animals were sacrificed at the appropriate time points by CO_2_ overdosage, followed by confirmatory cervical dislocation. Tumour tissues were excised at termination, snap frozen in chill isopentane and stored at −80 °C before further use.

### Tissue sectioning and mounting

LNCaP xenograft tumour was used for the MSI studies. Tissues were cut in a way that mimics a sagittal plane of slicing, as there is no spatial orientation with xenograft tumour. Cryosectioning was performed using a Leica cryotome (CM 1850 UV, Wetzlar, GmbH & Co. KG) at −18 °C and 12 μm tissue thickness. Sections were thaw-mounted onto conductive indium-tin-oxide (ITO)-coated slides (Bruker Daltonik, Bremen, GmbH & Co. KG), dried in a vacuum desiccator at room temperature for 30 min, and then stored at −80 °C until MSI analysis.

### Histological staining

See ESI† for method description.

### Off-tissue derivatization screening and TIMS separation

See ESI† for method description.

### OTCD reagent application for LNCaP xenograft tumour MALDI-2 assessment and imaging

From −80 °C, tumour tissue sections were dried in a vacuum desiccator (RT, 20 min). Gir-T and dansyl hydrazine reagents were dissolved at 0.1 mg mL^−1^ in 90 : 10 : 0.1 (v/v/v) methanol : water : trifluoroacetic acid (TFA) (10 mL) and were introduced to the ImagePrep® device (Bruker Daltonik, Bremen GmbH & Co. KG). A custom-edited method was created on the ImagePrep operating system as previously described by Smith *et al.*^17^ One-step was programmed to run for approximately 1 hour to achieve reaction completion. Conditions were as follows; ‘matrix thickness’ was manual set to 40 cycles, spray power at 25% and a fixed spray time of 2.2 s. Incubation time was set at 30 s with a fixed drying time of 60 s, which led to a final OTCD time of approximately 60 min per slide and a reagent density of 0.05 mg cm^−2^ for GT and 0.06 mg cm^−2^ for DS. The ImagePrep has an in-built sensor that is designed to monitor and control the thickness of MALDI matrices applied to sample slides. However, this feature was not used for reagent application.

### Matrix application

CHCA (6 mL, 5 mg mL^−1^ in 70 : 30 (v/v) acetonitrile : water + 0.1% v/v TFA) was applied in four passes using a modified 3D printer, as described in Tucker *et al.*^18^ A flow rate of 0.1 mL min^−1^ with a gas pressure of 2 bar, bed temperature of 30 °C, a *z*-height of 30 mm and stage velocity of 1100 mm min^−1^, averaging a run time of 24 min per slide. A uniform coating of matrix was achieved with a coverage of 0.11 mg cm^−2^. Matrix deposition conditions were evaluated—see ESI† for further details.

### MALDI-TIMS-tof MSI analysis

LNCaP mouse xenograft tumour and mouse prostate control tissues were analysed by MALDI-TIMS-tof MSI with and without laser-induced post-ionization for derivatized androgens. Data was acquired on a prototype timsTOF fleX MALDI-2 instrument (Bruker Daltonik, GmbH & Co. KG). MALDI was achieved with a Smartbeam 3D laser operating at 10 kHz for MALDI-1. Laser power was adjusted for each experiment to give the best resulting data. Laser focus was set to the minimum diameter and optimized to produce ablation craters of approximately 5 μm diameter. For imaging, pixel size was set to 50 μm in both *X* and *Y* axis and a beam scan of 46 μm, respectively, to investigate the full pixel area. Laser-induced post-ionization was achieved using a diode-pumped, actively Q-switched, frequency-quadrupled Nd:YAG laser operated at 1 kHz (NL 204-1k-FH, EKS-PLA, Vilnius, Lithuania) as described previously.^12^ The instrument was operated in positive ion mode, and ions between *m*/*z* 300 and 800 were accumulated from 769 laser shots per 50 μm pixel. For TIMS analysis, a ramp time of 769 ms was chosen including a range for 1/*K*_0_ from 1.1 to 1.75 Vs cm^−2^. Data analysis was performed with Data Analysis 5.3 (Build 342.363.6049, Bruker Daltonik, GmbH & Co. KG) and TIMS data viewer 1.0 (Build 0.164; Bruker Daltonik, GmbH & Co. KG). The resulting images were analysed using FlexImaging 5.0 (Build 80; Bruker Daltonik, GmbH & Co. KG) and SCiLS lab software (*vs.* 2021b; SCiLS Lab/Bruker Daltonik, GmbH & Co. KG).

### Confirmatory LC-MS/MS analysis

Confirmatory LC-MS/MS analysis was performed using a triple-quadrupole linear ion trap mass spectrometer (QTRAP 6500, AB Sciex, Cheshire, UK) coupled with an ACQUITY ultra high-pressure liquid chromatography system (UHPLC; Waters, Wilmslow, UK). See ESI† for further details.

## Abbreviations

MALDI: Matrix-assisted laser desorption/ionization
MALDI-2: Matrix-assisted laser desorption/ionization combined with laser-induced post-ionization
TIMS: Trapped ion mobility spectrometry
tof: Time-of-flight
OTCD: On-tissue chemical derivatization
CD: Chemical derivatization
MSI: Mass spectrometry imaging
T: Testosterone
DHEA: Dehydroepiandrosterone
CRPC: Castrate resistance prostate cancer
MS: Mass spectrometry
LC/MS: Liquid chromatography/mass spectrometry

## Author contributions

D. C., C. L. L. M., and J. S. conceived and coordinated the experiments. K. W. S., D. C., C. L. L. M., and J. S. designed the experiments. K. W. S., J. S., B. H., D. C. and C. L. L. M. carried out MS experiments. C. L. L. M., J. S., B. H., A. N.and D. C. conducted data processing and analysis. K. D. and J. S. provided equipment for MSI experiments. K. W. S., D. C., C. L. L. M., F. L. C., J. S. and K. D. wrote the manuscript, which was edited by all co-authors.

## Conflicts of interest

The authors declare the following competing financial interest(s): A. N. is an employee of Bruker Daltonik GmbH & Co. KG (Bremen, Germany); B. H. has been supported by Bruker Daltonik during the cause of the project. All other authors declare no conflict of interest.

## Supplementary Material

RA-011-D1RA06086D-s001
