# Supplementary material for: Spatial distribution of isobaric androgens in target tissues using chemical derivatization and MALDI-2 on a trapped ion mobility quadrupole time-of-flight instrument
Source: RSC Adv. 2021 Oct 18;11(54):33916–25. doi: 10.1039/d1ra06086d (PMC9042386; doi:10.1039/d1ra06086d)

Electronic Supplementary Material (ESI) for RSC Advances.
This journal is © The Royal Society of Chemistry 2021

Electronic Supporting Information
for
Spatial Distribution of Isobaric Androgens in Target Tissues Using Chemical Derivatization and MALDI-2 on a Trapped Ion Mobility Quadrupole Time-of-Flight Instrument

C.L. Logan Mackay[1], Jens Soltwisch[2], Bram Heijs[3,4], Karl Smith[5,&], Faye L. Cruickshank[1], Annika Nyhuis[6], Klaus Dreisewerd[2] and Diego Cobice[5]

[1] SIRCAMS, EastChem School of Chemistry, University of Edinburgh, Scotland, UK.
[2] Institute of Hygiene, University of Münster, Münster, Germany.
[3] Mass Spectrometry Imaging Group, Leids Universitair Medisch Centrum, Leiden, Netherlands
[4] Center for Proteomics & Metabolomics, Leiden University Medical Center, Leiden, Netherlands.
[5] Mass Spectrometry Centre, Biomedical Science Research Institute (BMSRI), Ulster University, Coleraine, Northern Ireland, UK.
[6] Bruker Daltonik GmbH & Co. KG, Bremen, Germany.

Corresponding author: Diego Cobice. d.cobice@ulster.ac.uk, tel: +44(0)2892604456

[&] Current address: National High Magnetic Field Laboratory (NHFML), Tallahassee, FL, USA.

Table of Content

1. **Supplementary Methods** **S2**
2. **Supplementary Figures** **S5**

1. Supplementary Methods

1.1 Histological staining

Cryosections were stained using haematoxylin and eosin. Tissue was fixated using cold acetone for 5 min, followed by a wash in sterile PBS (phosphate buffered saline) for 5 min. The sections were then dipped in haematoxylin for 20 s and placed under running water for bluing (15 min). Sections were then transferred to 0.25% eosin Y solution for 20 s, followed by five brief emersions in 100% ethanol and a 3 min wash in 95% ethanol. Stained tissue was mounted using Histoclear solution (NC9846201; Fisher Scientific International, Inc., Hampton, NH). Optical and magnified images of stained tissue were captured using an Epson V370 Photo scanner (Epson, Hemel Hempstead, UK) and a Zeiss Axio Scope.A1 (Zeiss, Jena, GmbH & Co K.G).

APES coating of histology slides

Briefly, 2% APES ((3-Aminopropyl) triethoxysilane in acetone) was used to immerse histology glass slides for 2 min, followed by two rinses in distilled H_2O, and allowed to dry at RT.

1.2 Off-tissue derivatisation reaction screening

Androgens working standards solutions (testosterone and DHEA) (10 ng/mL in methanol) were independently mixed in an Eppendorf tube with both Girard-T (0.1 mg/mL in 90:10 methanol: water (v/v) with 0.01 % trifluoroacetic acid (TFA)) or Dansyl Hydrazine (DS) (0.1 mg/mL in 90:10 Methanol: water (v/v) with 0.01 % (TFA) at a molar 1:2 ratio of androgen to derivatisation reagent. Each reaction was left for 1 hour at 40 ^{o}C. The reaction solution was then mixed with a molar 1:1 ratio of CHCA matrix (5 mg/mL 60:40 ACN:$H_2$0 (v/v) + 0.1% formic acid (FA)) and spotted onto a scout 384 stainless-steel MALDI target plate using the dried-droplet method for MALDI-TIMS-tof analysis.

Androgen mix (T+DHEA) solutions were prepared by mixing standard working solutions of both steroids in a 1:1 (v/v) ratio (5 ng/mL, each). Reaction, matrix addition, and MALDI spotting were carried out using both GT and DS reagents as previously described.[9]

1.3 Confirmatory LC-MS/MS of androgens in tissue homogenate

Stock androgen standards (T+DHEA) and internal standard (IS, d_3-testostereone) were prepared in methanol, with all stock concentrations at 1 mg/mL, with 99.9% purity certification. A working calibration curve was created by spiking an ethanol/acetone (1:1, v/v) mixture, with a concentration ranging from 0.1 – 1000 pg/g. A working IS solution was prepared containing 1 ng/mL, with each calibration standard spiked with 10 μL of working IS. Quality control (QC) standards were run using calibration points at 0.5 pg/g and 200 pg/g after 10 sample injections. All standards were stored at 2 – 8°C. This LC-MS/MS methodology was adapted from Cobice et al. [9] LNCaP tumour tissue (~50 mg) was homogenised in 700 μL of ethanol/acetone mixture (1:1, v/v) using ultrasonication device (UP 50H, MMTG, place, state) for 1 min at an amplitude of 80%, and 0.5-0.9 s intervals in a 1.2 ml Eppendorf. 20 μL of working internal standard at 1 ng/mL was spiked into the sample. The sample was kept in an ice bath during homogenisation. Then, 300 μL of MiliQ water was added to the mixture, vortexed for 30 s, ultrasonicated for 10 min, and centrifuged for 10 min at 15,000 rpm. The supernatant (600 μL) was taken and 10 mL of water was added prior to purification with a mixed-mode cation exchange cartridge (Oasis MCX 150 mg, 6 cc, Waters, Milton, MA). The cartridge was conditioned by loading with methanol and water (3 mL, each), then the sample was loaded and washed with 0.1 M HCl (1 mL) and water (3 mL). Androgens were eluted from the cartridge using acetonitrile/methanol (1:1, v/v, 4.5 mL). The supernatant was evaporated to dryness with nitrogen at RT and re-constituted in 200 μL of 50:50 (v/v) Mobile phase A: B using a 250 μL HPLC insert (1.5 ml HPLC amber vials). Samples were left at -20°C until LC/MS analysis. Homogenate samples were separated on a Luna PFP (50 x 2.1mm, 3.0 μm) column (Phenomenex, place, UK) at 40 °C on an Agilent HP 1250 UHPLC system (Waldbronn, GmbH & Co K.G) using acetonitrile with 0.01% (v/v) formic acid as mobile phase B and water with 0.01% (v/v) formic acid as mobile phase A. Starting gradient conditions were 40% B with 0.35 mL/min flow rate. The mobile phase gradient used is displayed in **Table 1**.

Table S1: Mobile phase gradient conditions for tumour homogenate androgen analysis

Time (min)	Mobile phase B (%)
0 – 6.0	40
6.0 – 12.1	90
12.1 – 15.1	40

Samples were kept at 5 °C and injection volume of sample was 10 μL. Mass spectrometry analysis was carried out using positive mode electrospray ionisation on a AB Sciex 6500 QTrap (AB Sciex, Concord, Canada). Mass spectrometry settings were tuned for maximum ion intensities for all analytes. Ionization was performed with instrument settings as follows: Dwell

time 150 ms, Source temperature: 550 °C, Ion source gas 1: 50 psi, Ion source gas 2: 50, Curtain gas: 30 psi, CAD gas: 12 psi, Ion spray voltage: 5500 V. Data were acquired by MRM (multiple reaction monitoring) transitions are detailed in **Table S2.**

Tables S2: Mass Spectrometry conditions

**Androgen**	**Parent (*m/z*)**	**Product (*m/z*)**	**CE (V)[1)]**	**DP (V)[1)]**	**CXP (V)[1)]**	**EP (V)[1)]**
T	289.2	109.0	33	90	10	8
DHEA	289.2	109.0	35	85	10	5
d_3-T (IS)	292.2	109.0	41	80	10	9

1) CE: Collision energy, DP: Declustering potential, CXP: Exit potential, EP: Entrance potential.

2 Supplementary Figures

Figure S1: Androgen Girard-T and Dansyl hydrazine derivatization reaction scheme

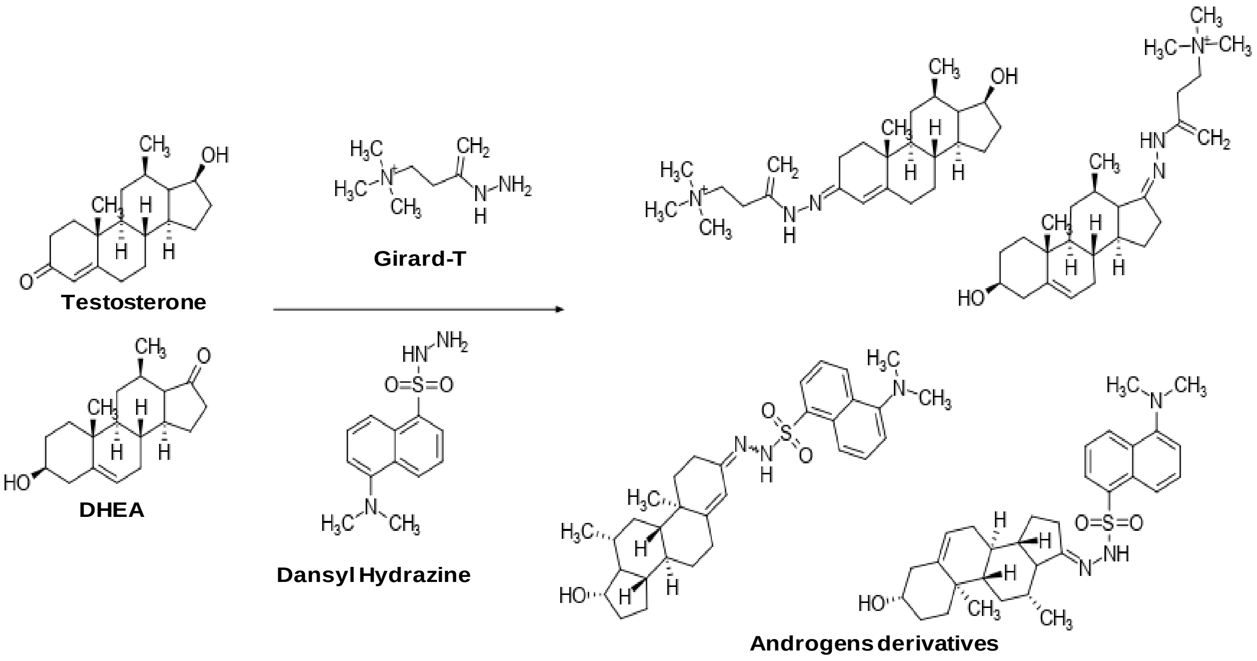

Figure S2: Representative off-tissue MALDI-TIMS-tof (10 scans accumulation) spectra of **a**) Girard-T testosterone derivative **b**) Girard-T DHEA derivative **c**) Dansyl hydrazine testosterone derivative **d**) Dansyl hydrazine DHEA derivative.

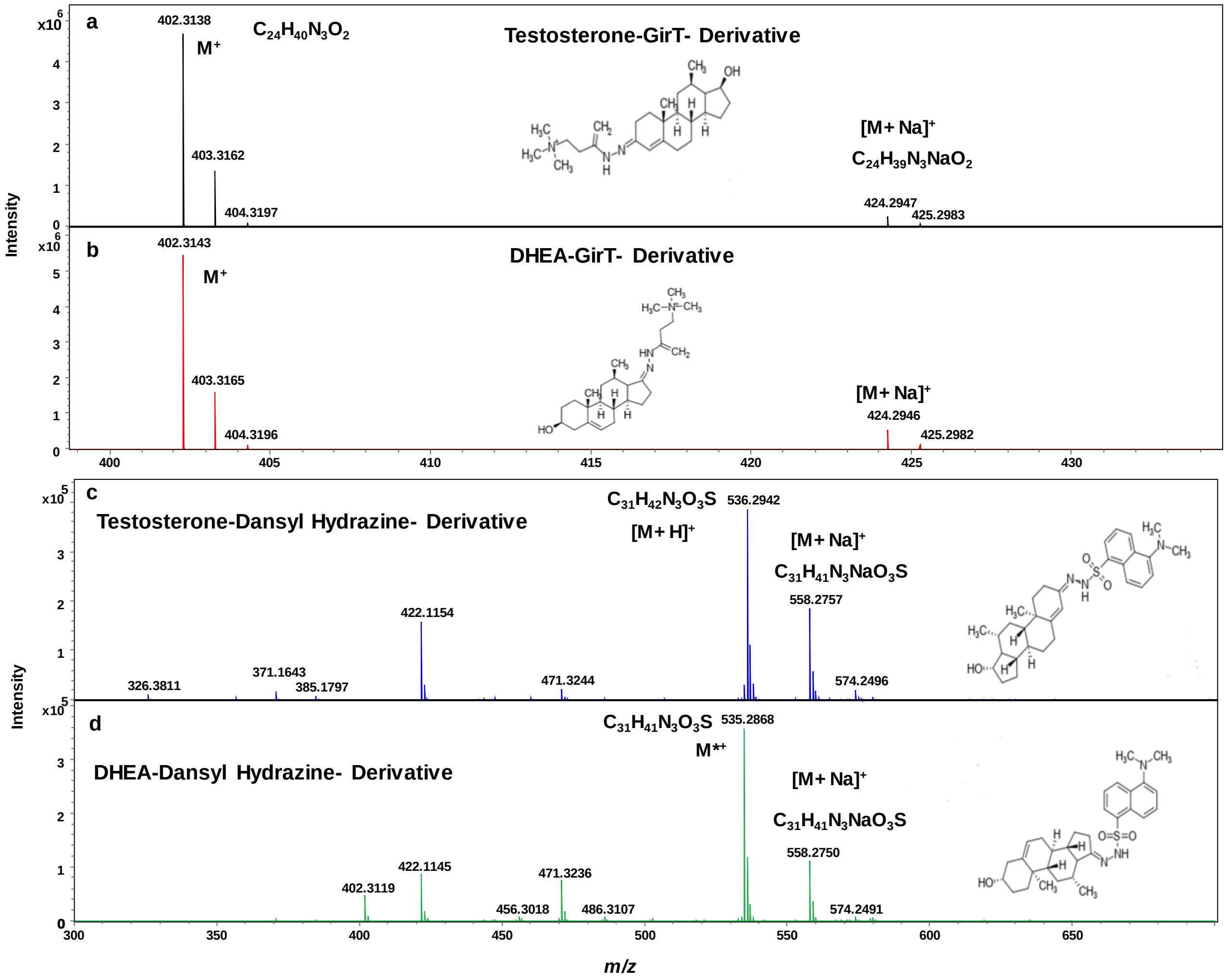

Figure S3: Representative off-tissue MALDI-TIMS-tof (10 scans accumulation) mobilograms of Girard-T testosterone derivative **a**) at its protonated mass **b**) at its sodium adduct. Girard-T DHEA derivative **c)** at its protonated mass **d**) at its sodium adduct. Equimolar solution of testosterone and DHEA Girard-T derivatives (5 ng/mL/each) at **e**) their protonated mass **f**) at their isobaric sodiated adduct **g**) Representative spectrum of equimolar solution of Girard-T derivatives (100 ng/mL/each)

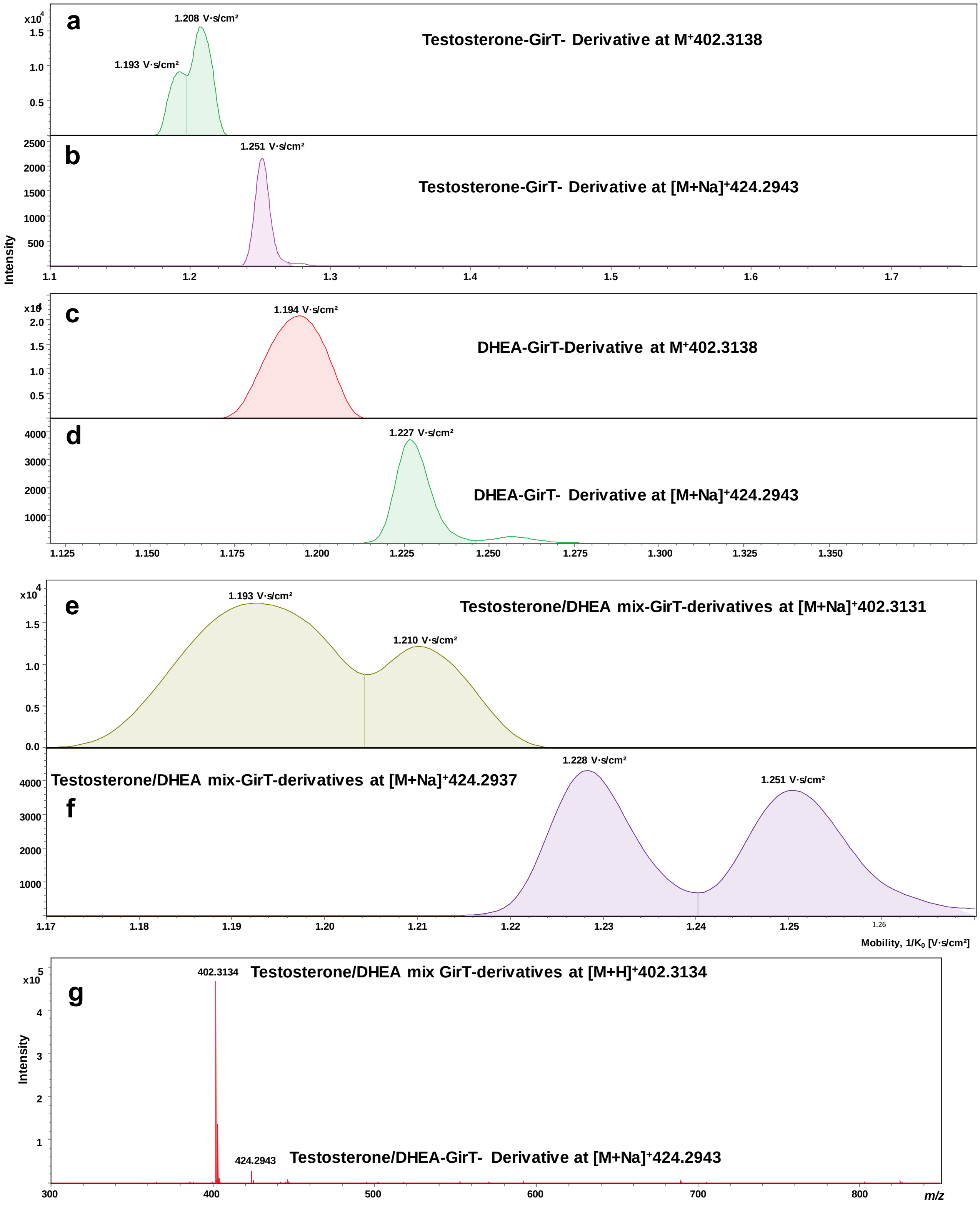

Figure S4: Representative off-tissue MALDI-TIMS-tof (10 scans accumulation) mobilograms of Dansyl-testosterone derivative **a**) at its protonated mass **b**) at its sodium adduct. Dansyl- DHEA derivative **c)** at its radical ion **d**) at its sodium adduct. Equimolar solution of dansyl testosterone and DHEA derivatives (5 ng/mL, each) **e**) at the protonated mass for testosterone **f**) at radical ion for DHEA **g**) at their isobaric sodiated adduct **h**) Representative off-tissue MALDI-TIMS-tof (10 scans accumulation) spectrum of dansyl testosterone + DHEA equimolar solution (100 ng/ml/each)

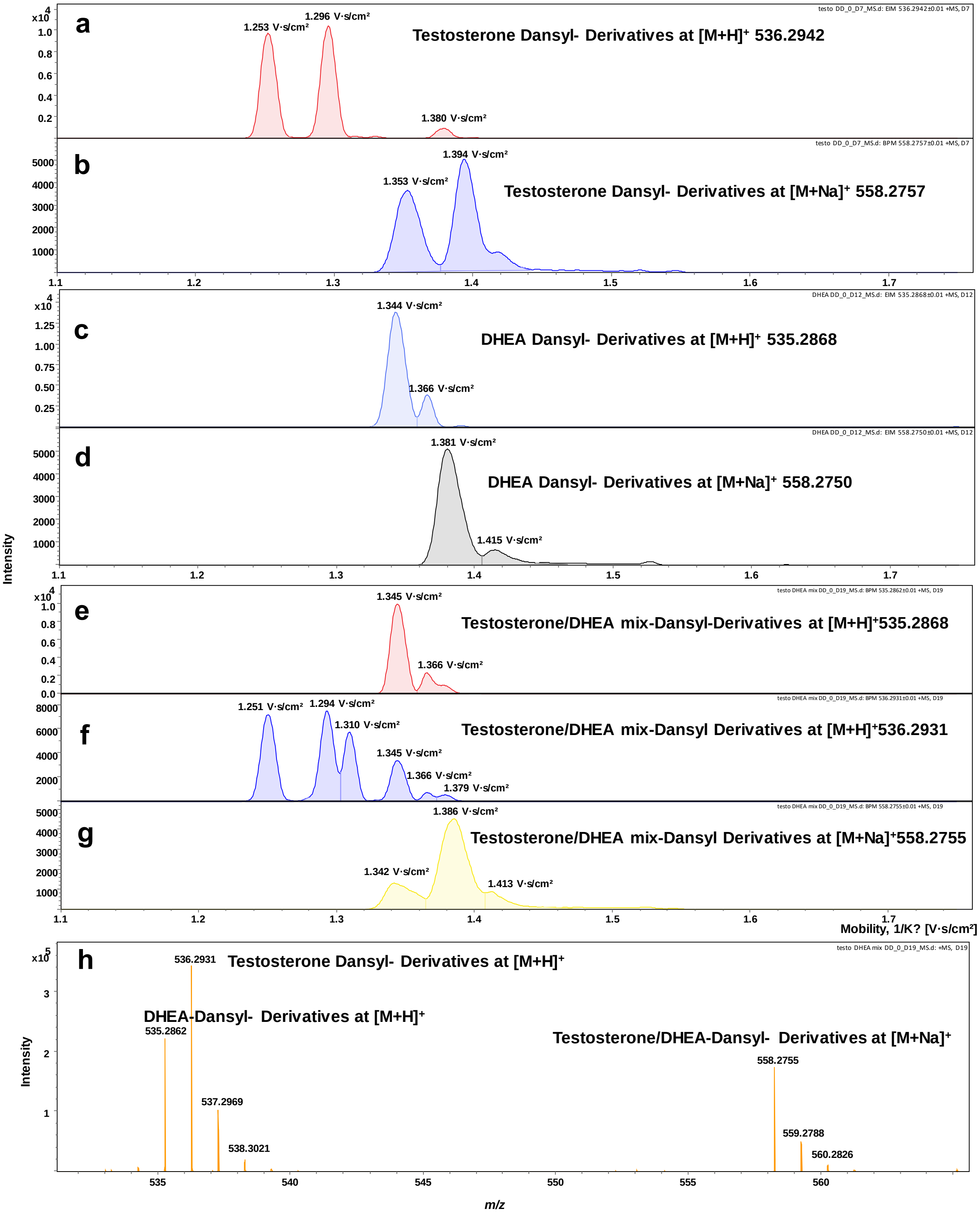

Figure S5: Quantitative and confirmatory LC-MS/MS analysis of testosterone and DEHA in LNCaP xenograph tumour. Tumour tissue homogenate was assessed for androgens levels **a**) Representative LC/MS/MS chromatogram of the targeted androgens in tissue homogenate sample.

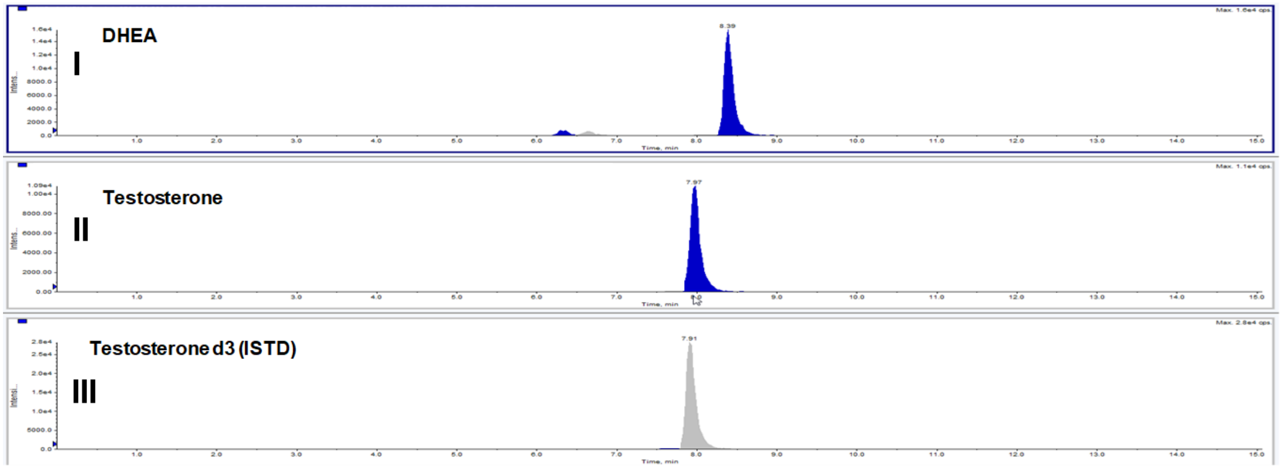

Supplement: RA-011-D1RA06086D-s001 [file RA-011-D1RA06086D-s001.pdf]
